# Formalin Fixation and Cryosectioning Cause Only Minimal Changes in Shape or Size of Ocular Tissues

**DOI:** 10.1038/s41598-017-12006-1

**Published:** 2017-09-21

**Authors:** Huong Tran, Ning-Jiun Jan, Danielle Hu, Andrew Voorhees, Joel S. Schuman, Matthew A. Smith, Gadi Wollstein, Ian A. Sigal

**Affiliations:** 1 Department of Ophthalmology, University of Pittsburgh, Pittsburgh, PA 15213 USA; 2 Department of Bioengineering, University of Pittsburgh, Pittsburgh, PA 15213 USA; 3 NYU Langone Eye Center, NYU School of Medicine, New York, NY 10016 USA

## Abstract

Advances in imaging have made it increasingly common to study soft tissues without first embedding them in plastic or paraffin and without using labels or stains. The process, however, usually still involves fixation and cryosectioning, which could deform the tissues. Our goal was to quantify the morphological changes of ocular tissues caused by formalin fixation and cryosectioning. From each of 6 porcine eyes, 4 regions were obtained: cornea, equatorial and posterior sclera, and posterior pole containing the optic nerve head. Samples were imaged using visible light microscopy fresh, 1-minute and 24-hours post-fixation, and post-cryosectioning. Effects were assessed by 14 parameters representing sample size and shape. Overall, formalin fixation and sectioning caused only minimal changes to the ocular tissues, with average percentage parameter differences of 0.1%, 1%, and 1.2% between fresh and post-fixing by 1 minute, 24 hours, and post-cryosectioning, respectively. Parameter changes were not directional, and were only weakly dependent on the duration of fixation and the region of the eye. These results demonstrate that formalin fixation and cryosectioning are good choices for studying ocular tissue morphology and structure, as they do not cause the large tissue shrinkage or distortions typically associated with other, more complicated, techniques.

## Introduction

*Ex-vivo* quantification and analysis of soft tissues benefits from histological processing techniques that prevent tissue degradation and preserve tissue morphology. Ideally, there would be no difference between *ex-vivo* tissues and their *in-vivo* counterparts. However, many imaging techniques require processing and labeling steps that can dehydrate and shrink the tissues^1–8^, thus potentially biasing measurements of tissues size, shape and microstructure.

Recent methods have been developed to quantitatively characterize soft ocular tissues without the need for dehydration or labels, reducing histological processing steps, for expediency, simplicity and to avoid potential artifacts. For example, small angle light scattering and wide angle x-ray scattering have been used to measure mean fiber orientation and dispersion without the need for labeling and do not necessarily require tissue fixation^9–11^. Another method that has gained in popularity is multiphoton microscopy, which allows visualizing collagen fibers in thick (up to 1 mm) sections or even uncut fixed tissues with high resolution. Multiphoton microscopy has been used to measure collagen orientation in posterior human scleral^12,13^, human cornea^14,15^, human lamina cribrosa^13^, rat scleral tissues^9^, all fixed with 2% or 4% paraformaldehyde and without any further histological processing steps. Additionally, we recently developed our own label-free method, polarized light microscopy^16,17^, that can quantify the micrometer-scale collagen fiber orientation of the ocular tissues.

Even though these techniques do not involve labeling or dehydration steps, they still often involve fixation and cryosectioning, which could potentially distort the tissue. Despite a substantial literature on this topic in other various soft tissues^1–5,18^, the effects of fixation and cryosectioning on ocular tissues have not been systematically quantified. Thus, the goal of our study is to quantify the changes in ocular tissue morphology, namely shrinkage and deformation, resulting from fixation and cryosectioning across a range of ocular tissues. For this study, we specifically test the effects of fixation using 10% formalin, a commonly used in the studies of ocular tissues^16,19–21^. To determine whether the effects of formalin fixation on ocular tissues are time dependent, we evaluate tissues after 1 minute and 24 hours of fixation. For our sectioning technique, we use cryosectioning in which the tissue is immersed in cryoprotectant, frozen and then cryosectioned. We hypothesize that formalin fixation and cryosectioning will preserve the size and shape of ocular tissues.

## Results

### Repeatability

All measurements have very good repeatability (Table 1). Coefficient of variations ranged from 0.1% to 1.1%, with an average of 0.4% across all parameters.

**Table 1:** Measurement repeatability.

Region	Parameter	Coefficient of variation (%)
Cornea	Area	0.3
Equatorial sclera	Area	0.4
	Length	0.2
Posterior pole containing optic nerve head	Area	0.3
Scleral canal	Area	1.1
	Diameter	0.2
Posterior sclera	Thickness	0.6
	Arc Length	0.1

### Fixative effects

The dimensional differences between fresh and fixed cornea, sclera, posterior pole containing the optic nerve head (PPONH), scleral canal, and scleral strip are small and difficult to discern visually (Fig. 2). Mean values and average percentage changes of each of 14 parameters at fresh, fixed 1 minute and fixed 24 hours were summarized in (Tables 2 and 3 **)**. The largest magnitude of percentage change, within each parameter, were also documented, as “worst cases” (Table 2). Overall, fixation effects were only significant for PPONH area (p = 0.0001), canal area (p = 0.002), canal NT diameter (p = 0.002), and no significance was found in other parameters (Table 4). Across all 14 parameters, average of mean percentage changes and maximum of absolute mean percentage changes were −0.1% and 1.3% from fresh to fixed for 1 minute, respectively, and −1% and 4.3% from fresh to fixed for 24 hours (Table 3).

**Figure 1:**
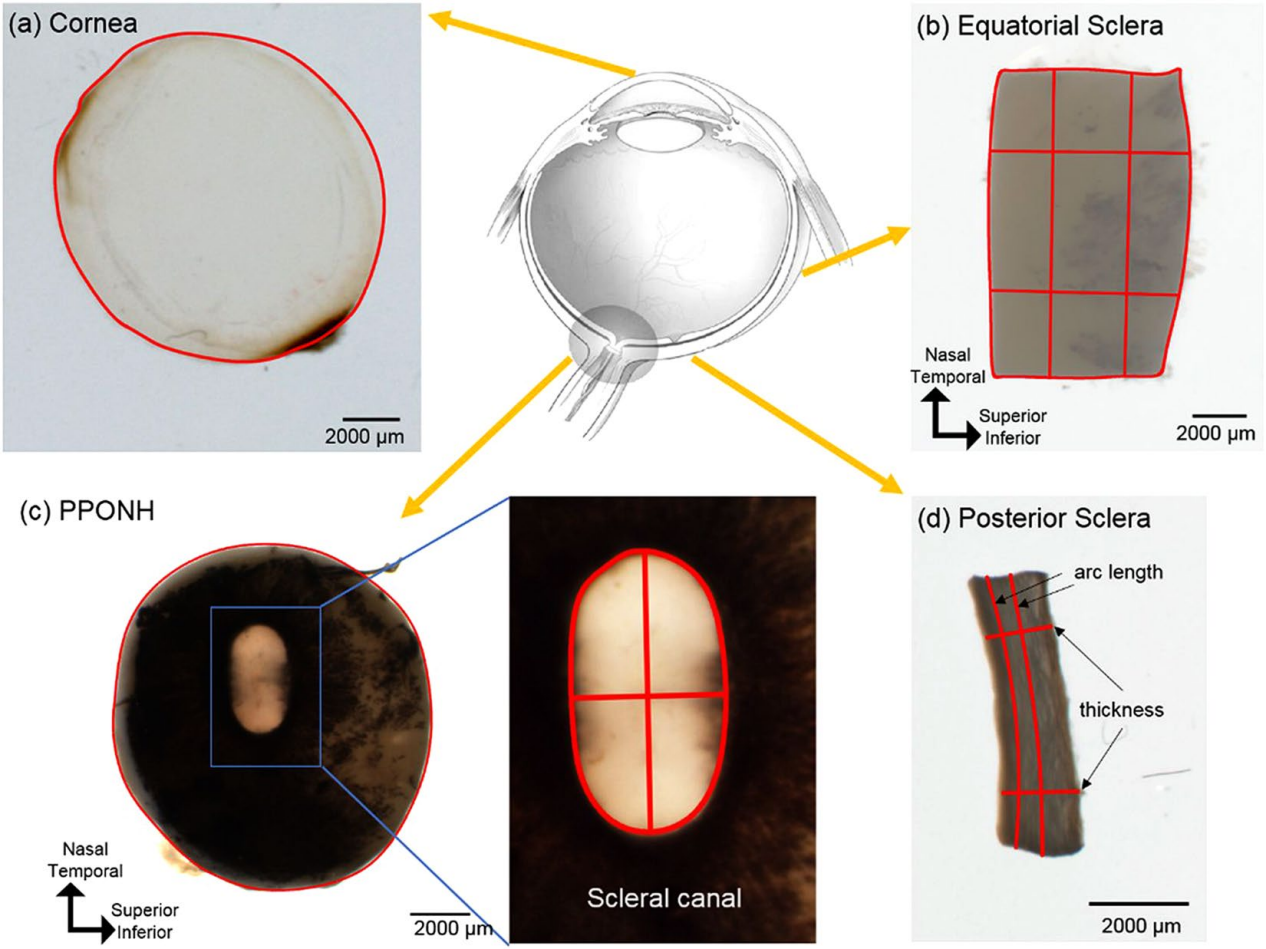
Example tissue samples with their corresponding markings for morphometry. From each whole fresh eye (top row center) (Adapted from Sigal *et al*.^38^), four regions were analyzed, including cornea (**a**), equatorial sclera as a rectangular piece (**b**), posterior pole containing the optic nerve head - PPONH (**c**), and posterior sclera strips (**d**). Areas were computed from the outline of the tissues (**a**–**c**). Measurements were taken separately of the ONH as a whole and of the scleral canal (**c**). In equatorial and posterior sclera (**b** and **d**), lengths and thickness were measured at two locations averaged.

**Figure 2:**
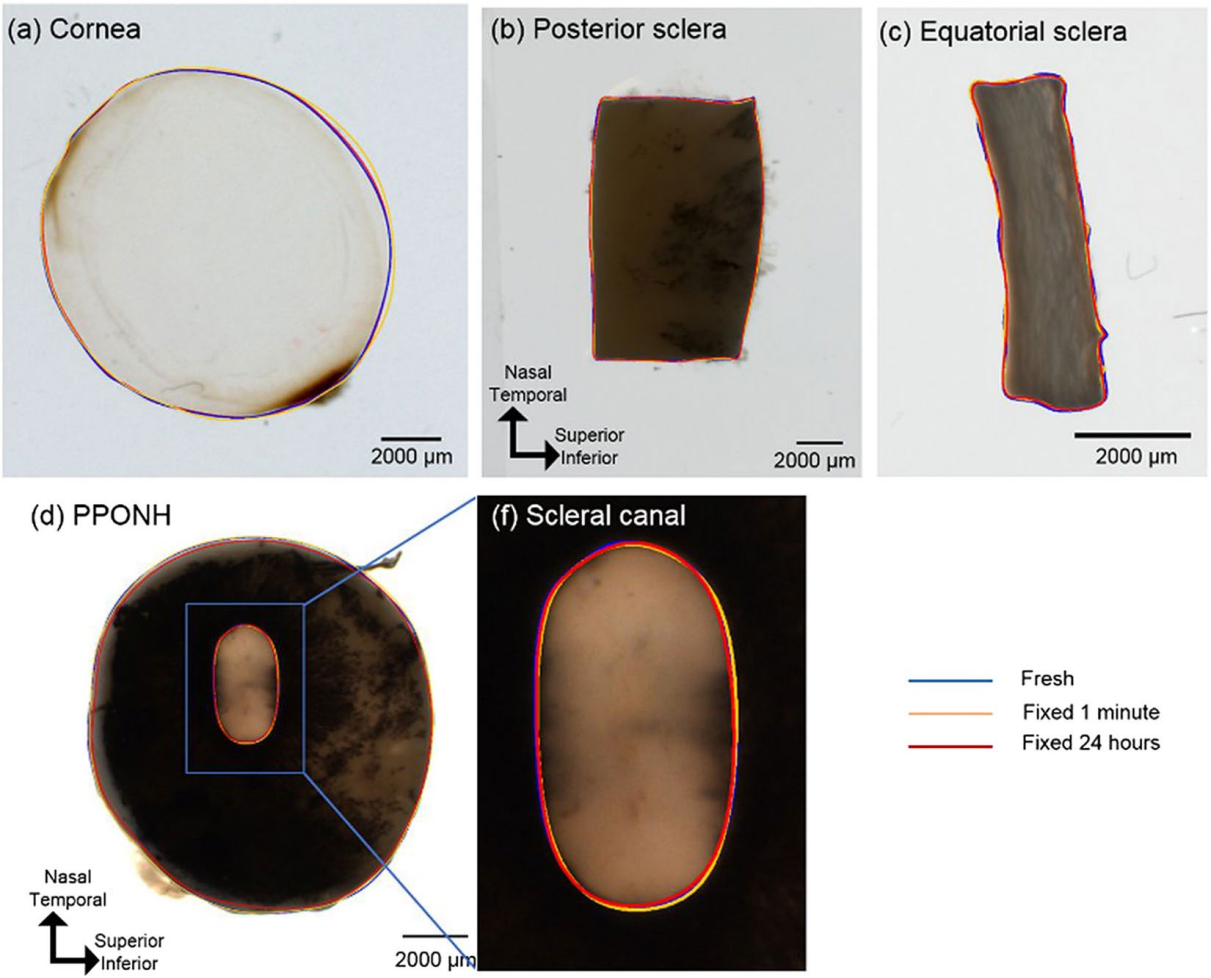
Images from fresh unfixed ocular tissues, overlaid with example manual markings from three time points: fresh (blue), fixed for 1 minute (yellow), fixed for 24 hours (red).

**Table 2:** Summary of mean values across 6 samples for each parameter at each time point (fresh, fixed 1 min, fixed 24 hours, and after sectioning).

Parameters		Mean values			
		Fresh	Fixed 1 min	Fixed 24 hrs	Sectioned
Cornea	Area (mm^2^)	92.8	93.5	95.3	—
	Circularity	0.99	0.98	0.99	—
Equatorial sclera	Area (mm^2^)	33.8	33.5	33.5	—
	Aspect ratio	0.56	0.55	0.55	—
	NT length (mm)	7.88	7.87	7.85	—
	SI length (mm)	4.32	4.29	4.27	—
Posterior sclera	Thickness (mm)	1.44	1.44	1.44	—
	Arc length (mm)	9.80	9.82	9.81	—
Posterior pole containing optic nerve head	Area (mm^2^)	97.9	98.0	95.6	96.6
	Circularity	0.996	0.996	0.995	0.996
Scleral canal	Area (mm^2^)	4.97	4.89	4.76	4.76
	Aspect ratio	0.59	0.60	0.59	0.58
	NT diameter (mm)	3.13	3.13	3.07	3.13
	SI diameter (mm)	1.86	1.88	1.81	1.84

**Table 3:** Summary of average percentage changes relative to fresh state of 14 parameters across 6 samples.

Parameters		Average of changes (%)			Worst cases (%)			Standard deviation of changes (%)		
		Fresh-Fixed 1 min	Fresh-Fixed 24 hr	Fresh-Sectioned	Fresh-Fixed 1 min	Fresh-Fixed 24 hr	Fresh-Sectioned	Fresh-Fixed 1 min	Fresh-Fixed 24 hr	Fresh-Sectioned
Cornea	Area	0.6	2.5	—	2.2	8.2	—	0.9	2.6	—
	Circularity	−0.2	−0.1	—	−1.1	−0.7	—	0.5	0.3	—
Equatorial sclera	Area	−0.6	−0.8	—	−2.6	−2.1	—	1.2	1.2	—
	Aspect ratio	−0.6	−0.8	—	−1.4	−2.6	—	0.6	0.9	—
	NT length	−0.01	−0.3	—	−1.1	−1.1	—	0.5	0.5	—
	SI length	−0.6	−1.0	—	−2.5	−3.0	—	0.7	1.0	—
Posterior sclera	Thickness	−0.4	−0.5	—	−2.3	−3.4	—	0.6	1.4	—
	Arc length	0.1	0.01	—	−1.0	−0.8	—	0.3	0.3	—
Optic nerve head	Area	0.1	−2.4	−1.2	−1.1	−3.5	−6.5	0.7	0.6	3.0
	Circularity	0.002	−0.1	−0.02	−0.1	−0.2	−0.1	0.0	0.1	0.1
Scleral canal	Area	−1.3	−4.3	−3.4	−4.8	−6.3	−7.7	1.6	1.7	3.7
	Aspect ratio	1.0	−0.8	−1.6	2.4	−3.7	7.5	0.8	1.9	5.2
	NT diameter	−0.3	−2.2	−0.1	−1.6	−3.7	4.2	0.8	1.1	2.0
	SI diameter	0.7	−3.0	−0.9	1.1	−6.1	−7.7	0.4	2.4	4.8
*Across* 14 *parameters*	*Average* (%)	***−0.1***	***−1.0***	***−1.2***						
	*Maximum of absolute* (%)	***1.3***	***4.3***	***3.4***						

Within each parameter, the largest magnitude of percentage change, either worst cases in positive or negative direction, and the standard deviation of percentage changes are listed. Average changes and maximum of absolute changes across 14 parameters are also summarized.

**Table 4:** ANOVA results of measurements across 3 time points, fresh, fixed 1 minute, and fixed 24 hours.

Parameters		p-value
Cornea	Area	0.10
	Circularity	0.54
Equatorial sclera	Area	0.29
	Aspect ratio	0.08
	NT length	0.29
	SI length	0.06
Posterior sclera	Thickness	0.67
	Arc length	0.42
Posterior pole containing optic nerve head	Area	0.0001*
	Circularity	0.03
Scleral canal	Area	0.002*
	Aspect ratio	0.10
	NT diameter	0.002*
	SI diameter	0.02

*Significant level p = 0.0025.

### Fixative effects-Across eye regions

**Figure 3:**
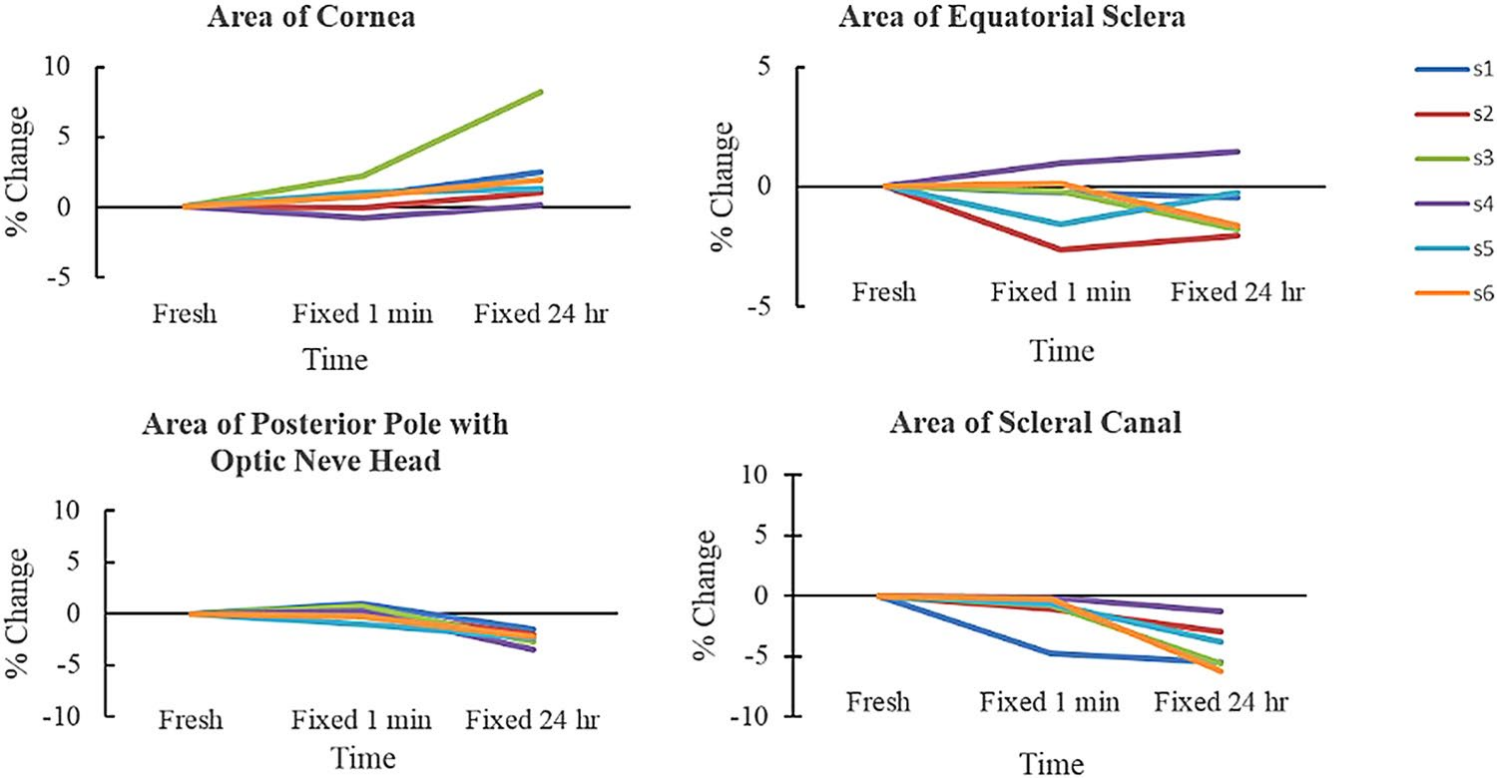
Regional-dependent tissue area changes of ocular tissues across 3 time points: fresh, fixed 1 minute, and fixed 24 hours. Each line corresponds to one tissue sample. All values are changes relative to the fresh sample baselines.

On average, fixing for 1 minute and 24 hours increased cornea area by 0.6% and 2.5%, respectively (Fig. 3, Table 3). There was an extreme case in which the cornea area increased up to 8.2% after fixing for 24 hours. Areas of equatorial sclera decreased very slightly after fixation, with average percentage decreases less than 1%. PPONH areas had average percentage changes of 0.1% and −2.4% after fixing for 1 minute and 24 hours, respectively (Fig. 3, Table 3). Specifically, PPONH area decreased significantly by 2.35 mm^2^ or 2.4% between fresh and fixed for 24 hours (p = 0.0002), and decreased significantly by 2.44 mm^2^ or 2.5% between fixed for 1 minute and fixed for 24 hours (p = 0.0014) (Table 5). The scleral canal area decreased significantly by 0.13 mm^2^ or 2.6% between fixed for 1 minute and fixed for 24 hours (p = 0.005).

**Table 5:** Paired t-tests of fixative effects across 3 time points, fresh, fixed 1 minute, and fixed 24 hours.

Parameters		Mean differences			p-value			Normalized mean differences (%)		
		Fresh-Fix 1 min	Fresh-Fix 24 hrs	Fix 1 min-Fix 24 hrs	Fresh-Fix 1 min	Fresh-Fix 24 hrs	Fix 1 min-Fix 24 hrs	Fresh-Fix 1 min	Fresh-Fix 24 hrs	Fix 1 min-Fix 24 hrs
Posterior pole containing optic nerve head	Area (mm^2^)	0.09	**−2**.35*	**−2**.44*	0.7778	**0**.0002*	**0**.0014*	0.1	**−2**.4*	−2.5*
Scleral canal	Area (mm^2^)	−0.08	−0.20	**−0**.13*	0.1931	0.0058	**0**.0050*	−1.6	−4.1	−2.6*
	NT diameter (mm)	−0.01	**−0**.07*	−0.06	0.5220	**0**.0051*	0.0136	−0.2	−2.1*	−1.9

*Significant level p = 0.0056.

### Fixative effects-Across different directions

**Figure 4:**
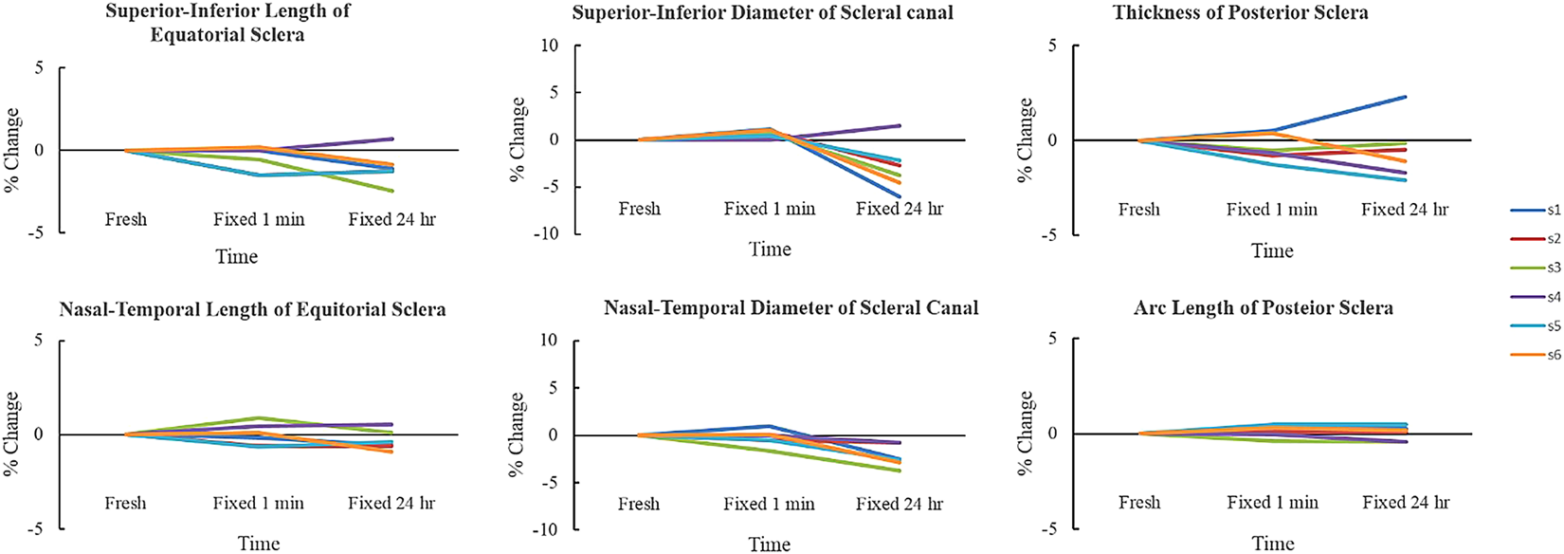
Dimensions of ocular tissues across 3 time points: fresh, fixed 1 minute, and fixed 24 hours. Each line corresponded to one tissue sample.

Both equatorial scleral lengths in nasal-temporal and superior-inferior directions did not decrease very much after fixation, with all average percentage decreases less than 1% (Fig. 4, Table 3). The canal nasal-temporal diameter decreased significantly by 0.07 mm or 2.1% between fresh and fixed for 24 hours (p = 0.0051) (Table 5). Scleral canal’s superior-inferior diameter changed on average by 0.7% and −3% after fixing for 1 minute and 24 hours, respectively (Fig. 4, Table 3).

**Figure 5:**
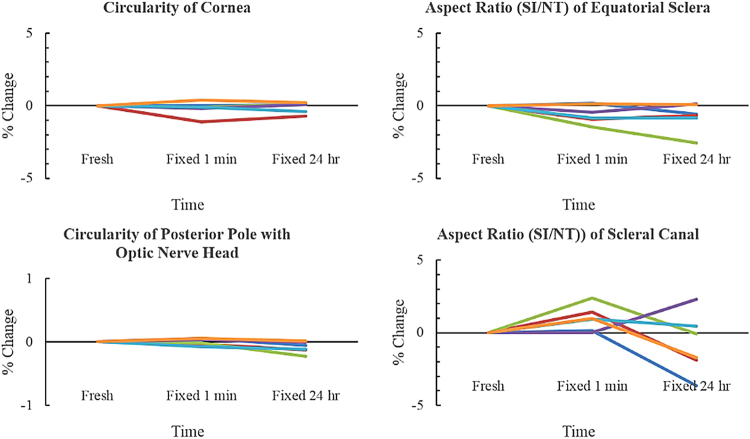
Circularity and aspect ratio of ocular tissues across 3 time points: fresh, fixed 1 minute, and fixed 24 hours. Each line corresponded to one tissue sample.

Circularity of the cornea did not change much after fixation, with average percentage changes less than −0.2% and the worst case had percentage changes of −1.1% from fresh to fixed for 1 minute (Fig. 5, Table 3). Similarly, circularity of the PPONH did not change much after fixation, with average percentage changes of 0.002% and −0.1% after fixing for 1 minute and 24 hours, respectively. Aspect ratio of the equatorial sclera decreased on average by 0.6% and 0.8% after fixing for 1 minute and 24 hours, respectively (Fig. 5, Table 3). Aspect ratio of the scleral canal also had very small changes, increasing by 1% and decreasing by 0.8% after fixing for 1 minute and 24 hours, respectively, with the worst case up to −3.7% after fixing for 24 hours.

For the posterior sclera, the thickness (width in the out of plane direction), decreased by 0.4% and 0.5% after fixing for 1 minute and 24 hours, respectively (Fig. 5, Table 3). The arc length, or the in-plane direction, also had very small changes, with average percentage increases of 0.1% and 0.01% after fixing for 1 minute and 24 hours, respectively.

**Figure 6:**
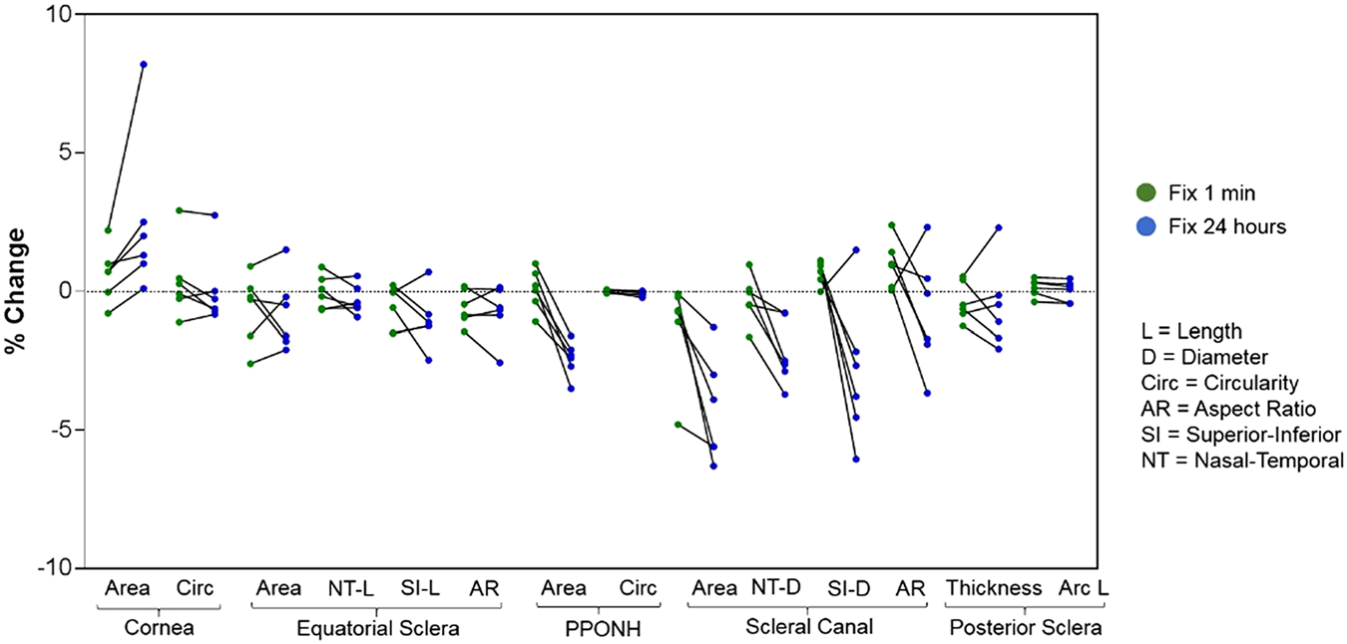
Percentage changes in sample dimensions relative to fresh after formalin fixation for 1 minute (green dots) and 24 hours (blue dots).

To simplify comparison of the effects of fixation time across all 14 parameters, the percent changes relative to fresh were plotted in a single figure (Fig. 6).

### Sectioning effects

**Figure 7:**
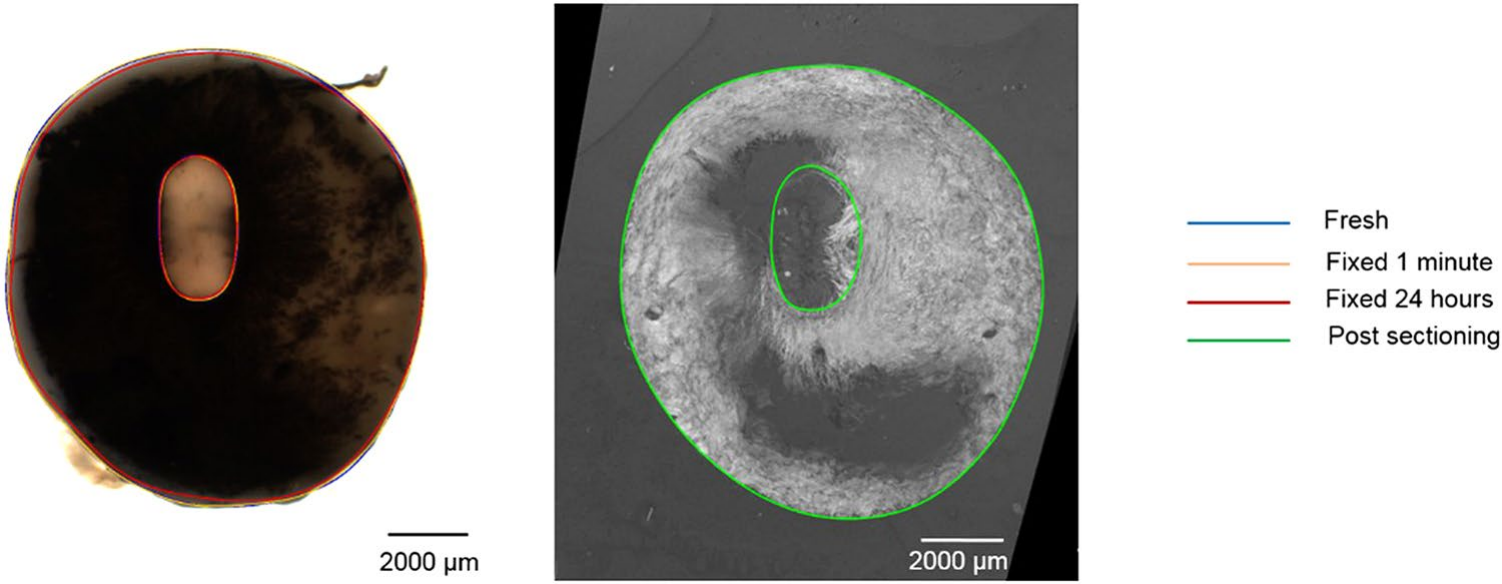
Example comparison of a fresh PPONH (Left) with a post-sectioning PPONH (Right). The post-sectioning PPONH is a projection of 3 adjacent histological sections.

**Figure 8:**
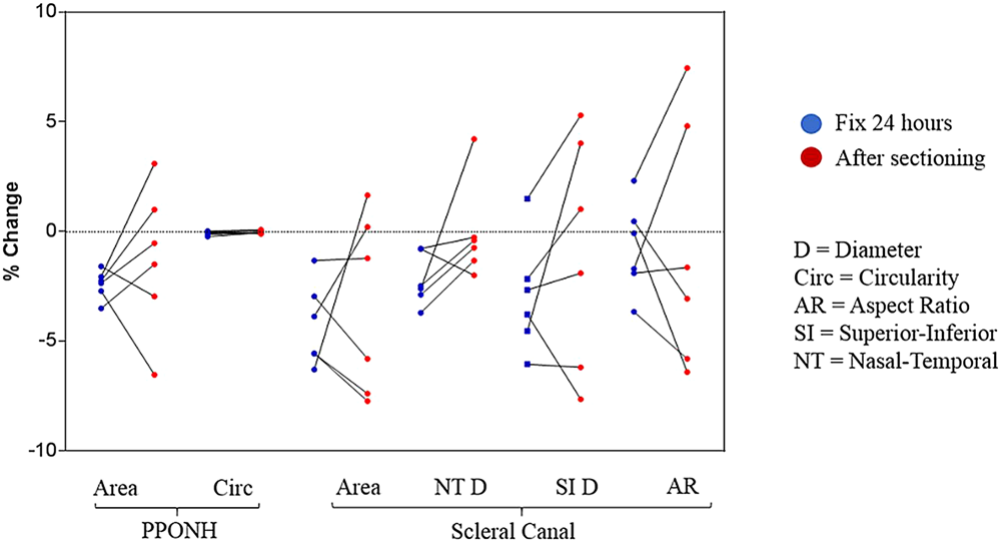
Summary of effect of 24-hour formalin fixation (blue dots) and sectioning process (red dots) on the sizes of PPONH and scleral canal.

Visually, the disparities between fresh and histological (post-sectioning) were minor, in terms of shape, size or aspect ratio (Fig. 7). Compared with the changes after 24-hour fixation, the percentage changes after histological processing were more varied (Fig. 8). Mean values and average percentage changes of the PPONH and the scleral canal measurements at fixed 24 hours were summarized in (Tables 2 and 3). Overall, there was no statistically significant difference detected among measurements across 4 time points (fresh, fixed 1 minute, fixed 24 hours, post sectioning) for all 6 parameters of the PPONH and the scleral canal (Table 6).

**Table 6:** ANOVA results comparing measurements across 4 time points: fresh, fixed 1 minute, fixed 24 hours, and post sectioning.

Parameters		p-value
Posterior pole containing optic nerve head	Area	0.16
	Circularity	0.07
Scleral canal	Area	0.01
	Aspect ratio	0.47
	NT diameter	0.03
	SI diameter	0.21

*Significant level p = 0.0025.

Across all 6 parameters, average of mean percentage changes and maximum of absolute mean percentage changes were −1.2% and −3.4% from fresh to post sectioning (Table 3). Post sectioning, the PPONH area decreased on average by 1.2%, whereas the scleral canal’s area decreased by 3.4%. Both the PPONH circularity and scleral canal’s aspect ratio had very small changes, with an average percentage changes of −0.02% and −1.6%, respectively. The canal superior-inferior diameters changed in their sizes slightly more than those from the canal nasal-temporal diameters, however, these differences were not large.

## Discussion

Our goal was to systematically quantify the morphological changes in ocular tissues resulting from formalin fixation, for 1 minute and 24 hours, and the process of tissue preparation and cryosectioning. 1 minute fixation was used to quantity the immediate effect of formalin fixation whereas 24 hours has been recommended for thorough tissue fixation^22,23^. The last time point, post-sectioning, was used to quantify the effect of histological processing process. Measurements’ repeatabilities are good, with coefficients of variation two to three orders of magnitude smaller than the parameters. We found that fixation caused only minimal changes in tissue dimensions, with average changes under 1%. Fixation-induced changes are only statistically significant for three parameters: the PPONH area (p = 0.0001), the scleral canal’s area (p = 0.002) and the nasal-temporal diameter (p = 0.002), which had average changes under 2.6%. Additional tissue processing and sectioning cause further dimensional changes, but these are still generally small, −1.2% on average. As expected, there was substantial variability in the changes with fixation and sectioning. The largest “worst case” fixative effect was an 8.2% increase in cornea area between fresh and fixed for 24 hours. The largest sectioning effects were 7.7% decreases in scleral canal area and superior-inferior canal diameter between fresh and sectioned tissues.

When comparing across all samples, there are no strong or consistent trends for parameter changes, except for the cornea area. Cornea areas of all samples increased consistently with formalin fixation by an average of 0.6% after fixing 1 minute and 2.5% after fixing for 24 hours. This could be due to swelling or edema, known to be substantial in cornea *ex-vivo* despite the tissues being kept in PBS or fixative^24^. These results are important because they establish that formalin fixation and further histological processing for cryosectioning maintain the overall shape and size of ocular tissues. Fixing and sectioning tissues is indispensable in many research activities, and it is therefore necessary to ascertain its effects.

Our results of minimal change in ocular tissue dimensions with formalin fixation were similar, and sometimes smaller than values reported in the literature. Temporal arteries were reported to have 8% or an average of 2–4 mm shrinkage and sometimes up to 20% shrinkage post formalin fixation^6,25^. Prostate tissues were found to lose 5.8% in their weight after immersing in 10% formalin^26^. Shrinkages had been measured to be 2.7% in cervical tissue post 8% formalin fixation for 24 hours^1^, 4.6% in tumor tissue post 10% formalin fixation for 6–24 hours^7^, 1–8% in human brain stem post 4% formalin fixation^2^, and 4.1% in prostate cancer tissue post 10% formalin fixation for 24 hours^8^. The study of prostate cancer tissues also reported dimensional changes post fixation due to additional histological processing of up to 15%^8^, which was larger than what we found. This could be due to differences in histological processing, including tissue embedding medium or cryosectioning’s thickness, temperature, or tissue types.

The dimensional changes measured in this study were generally smaller than those reported using other fixatives and histological processing. For example, a combination of either formalin, glutaraldehyde, or McDowell’s solution with paraffin embedding caused up to 19–25% shrinkage in cross-sectional area of arterial rings^5^. In ocular tissues, Roberts and colleagues reported using a “correction” scaling factor of 1.7–2.5 to account for shrinkage caused by the tissue processing used for 3D reconstruction of monkey ONH and sclera^27^. Other studies evaluated the effects of fixation on tissue integrity and gross curvature of the whole globe using various concentrations of glutaraldehyde and formaldehyde, reporting sometimes substantial effects of fixation^28,29^.

Cryosectioning is becoming increasingly common with the advent of imaging techniques that enable visualization of ocular tissues without the need for tissue dehydration, staining and/or labeling, such as second harmonic generation^30^ and polarized light microscopy^16^. This is a great advantage of the technique, since the use of stains or labels as well as ethanol dehydration, and embedding into plastic or paraffin are known to cause tissue shrinkage and deformation. For example, cervical tissues that went through 8% formalin fixation for 24 hours, with additional dehydration and paraffin wax embedding had a total shrinkage of approximately 15%^1^. Similarly, human brain stem had up to 17% shrinkage in size after paraffin embedding^2^. It is important that we found that both shape and size were only minimally affected by fixation and sectioning. We have shown elsewhere that formalin and cryosectioning can also produce excellent images with polarized light microscopy^16,17^, without the artifacts of fixation and paraffin of plastic embedding. Other techniques could privilege one at the expense of the other. For example, block-face imaging methods that minimize deformation artifacts due to sectioning have been developed and applied to the eye^31^, but the embedding techniques involved cause substantial tissue shrinkages, sometimes over 60%^27^.

Histological processing involves several stages, including cryoprotecting, sectioning, imaging, image-stacking, each of which can introduce deformations and artifacts. A strength of our methodology is that we were able to ascertain the combined effects of all these steps, and also isolate them from the effects of fixation. Another strength of this study is that we analyzed tissues from several regions of the globe, and studied them using a several parameters representing both shape and size. Thus, we were able to obtain a more comprehensive assessment of the effects of the fixative and sectioning.

Studies in other tissues and processing techniques have found that fixation times can have substantial effects on the samples^22,23^. Hence, we opted to test whether fixing for a very short 1 minute would have different effects than fixing for 24 hours. Potential effects of fixation time could also be important when fixing large or thick samples due to fixative perfusion and penetration, or when tissues are fixed by perfusion rather than immersion. Our results are therefore also important, because they show that differential deformation and/or shrinkage due to penetration or perfusion variations are also likely small. This is consistent with previous findings on prostate cancer tissues^26^.

Our measurements post-sectioning were based on 3D stack reconstructions with maximal intensity projections. This technique was developed to minimize errors caused by tissue tilting or deformation during sectioning. To the best of our knowledge, most morphometry-based studies do not take into account the 3D nature of the tissues and are therefore subject to errors due to sectioning direction, which would compound and affect morphometry results. Still, it is important to acknowledge that we used manually placed outlines and that the image projections for measurement were approximations.

There was more variability in the percent changes across samples after sectioning than after fixation. This is not surprising considering the potential for artifacts from this and later image processing, as mentioned above. This variability likely contributed to the fact the significant ANOVA results among the first 3 time points for PPONH area, scleral canal’s area and nasal-temporal diameter were no longer significant when testing across 4 time points. This suggest that the variability induced by histological processing and sectioning should be considered as likely more substantial than that from shrinkage when interpreting data from ocular tissues fixed with formalin. In terms of sectioning, in this study we only assessed the combined effects caused by sucrose cryoprotecting, freezing, cryosectioning, imaging, image processing, stacking and measurement. We did not isolate the effects of each step. Our results might be limited to this particular histological procedure, and additional tests will be needed for other procedures.

We only tested shrinkage effects on porcine eyes and did not test for possible shrinkage differences across species, i.e. nonhuman primate or human. However, porcine eyes are often considered to be very similar to human eyes on several aspects, especially the existences of well-defined scleral canal with a collagenous lamina cribrosa, a structure that usually does not exist in lower level species^32^. It might also be important in future studies to account for possible covariant factors that could affect shrinkage, such as age. In this study we only studied excised fresh ocular tissues, and each ocular region was fixed separately instead of perfusion fixing the whole eye globe. This could be important due to residual deformations^33^.

We note that our goal in this study was not to compare formalin fixation and cryosectioning with other techniques, such as paraffin or plastic embedding, or sectioning with a vibratome. Formalin fixation and cryosectioning are of increasingly common use in the study of ocular tissues, and it is therefore essential to determine their effects on the tissues size and shape. Future studies could address other important questions, such as the effects of formalin fixation on cellular structures, immunoreactivity, or on the ability to extract nucleic acids or protein from ocular tissues. Considering the small sample set, if precise estimates of fixative or cryosection effects are needed by future studies, a larger sample set should be analyzed.

In summary, formalin fixation and sectioning caused minimal changes in the size or shape of ocular tissues, with decreases with respect to the fresh tissues averaging 0.1%, 1%, and 1.2% post fixing 1 minute, 24 hours, and sectioning, respectively. These morphological changes were not dependent on the direction (superior-inferior versus nasal-temporal, in-plane versus out-of-plane), and were weakly dependent on the duration of fixation (slightly larger effects after 24-hour fixation than after 1 minute) and the regions of the eye (cornea, equatorial scleral, PPONH, scleral canal).

## Materials and Methods

### General Approach

Six fresh porcine (*sus scrofa domesticus*) eyes ranging from 6 to 8 months old were obtained from the local abattoir and processed within 2 hours post death. Samples were taken from the cornea, sclera, and PPONH, and sclera (Fig. 1) and imaged using bright field microscopy to establish the fresh condition. The samples were then imaged after 1 minute and after 24 hours of fixing in 10% formalin. The 24-hour fixed PPONH samples were also sectioned and the sections imaged again. Below we describe the steps in more detail.

### Fresh tissue preparation

An 11 mm circular trephine was used to excise samples from the cornea and the posterior pole containing the ONH and surrounding sclera tissues, the PPONH. Scissors and scalpels were used to cut rectangular samples of posterior and equatorial sclera. The fresh tissue samples were then placed in petri dishes and kept moist with phosphate-buffered saline-PBS (Fisher Scientific, Pittsburgh, PA) for imaging as described below.

### Formalin fixation and histological processing

All fresh tissues were immersion-fixed by rapidly replacing the PBS with 10% formalin (Fisher Scientific, Pittsburgh, PA) for 1 minute at room temperature, taken out briefly for imaging and then returned to fixative for 24 hours. To study the effects of sectioning, the 24-hour fixed PPONHs were further processed, including immersing in a 30% sucrose solution for cryoprotection and embedding in Optimal Cutting Temperature compound (Fisher Scientific, Pittsburgh, PA) before freezing in liquid nitrogen (−196 °C). Frozen blocks of PPONH tissue were then cryosectioned coronally at 30 µm thickness (Leica CM3050 S, Leica Biosystems Inc., Buffalo Grove, IL).

### Imaging and 3D reconstruction of histological images

All samples were imaged using standard white light microscopy in a dissecting microscope (SZX16, Olympus, Tokyo, Japan) when fresh, after 1 minute and after 24 hours immersed in the 10% formalin fixative. Morphometry requires clearly discerning the tissues, but this is sometimes not trivial in unstained cryosections. Rather than staining the fixed PPONH sections, which could potentially alter the tissue dimensions, high-contrast images were obtained by using polarized light microscopy (12-bit greyscale, 0.5x objective lens, 0.07 NA, 1x magnification setting) as described elsewhere^16^. To allow fair comparison between tissues before and after cryosectioning, the set of section images for an PPONH were assembled into a 3D stack by manually registering sequential section images based on PPONH anatomical landmarks, including scleral canal, central retinal vessels, and major LC collagen beams using methods previously reported^34^.

### Markings & Repeatability

Sizes and shapes of the tissue samples were manually determined using the FIJI software package^35^. All measurements were performed by one observer and the repeatability was intra-observer. Specifically, 14 parameters were quantified: area and circularity of the cornea; area, lengths (SI: superior-inferior, NT: nasal-temporal), and aspect ratio of the equatorial sclera; area and circularity of PPONH; area, diameters (SI, NT), and aspect ratio of the scleral canal; thicknes and arc length of the latitudinal posterior sclera strip (Fig. 2). Anisotropy of tissue changes were also quantified using circularity and aspect ratio. Circularity was computed using ImageJ, defined as

1 $${\rm{Circularity}}=4{\rm{\pi }}\times ({\mathrm{Area}/\mathrm{Perimeter}}^{2})$$

with a circularity of 1 representing a perfect circle, and smaller values representing a less circular, elongated, shape. Aspect ratios of the equatorial sclera and the scleral canal were calculated as the ratio of superior-inferior length over nasal-temporal length. The outlines of the cornea and PPONH were marked as polygons. Arc lengths of the posterior sclera were measured in the latitudinal direction, and thicknesses were marked as the distances between two locations connecting the interior and exterior sides. Measurement locations were selected such that they were easily discernible across all time points.

To assess repeatability, a set of 3 repeated markings on a randomly selected sample at random time point was collected for cornea area, equatorial sclera’s area and length, PPONH’s area, scleral canal’s area and diameter, and posterior sclera’s thickness and arc length. Repeatability was determined by coefficient of variation (CV),

2 $${\rm{CV}}( \% )=\frac{{\rm{\sigma }}}{\,\mu }\times 100$$

in which σ (µm) is the standard deviation and μ (µm) is the mean across repeated markings.

All measurements acquired before sectioning were made on 2D images of 3D samples, thus these images are 2D “projections” of the 3D tissue as visible through the microscope. To fairly compare with the 3D stacks assembled from sections, we also computed projections of the 3D stacks. For the outline of the PPONH region, the entire stack of images for an eye was projected in the anterior-posterior direction using maximal intensity. For the scleral canal outline in the histology stack the canal outline was obtained from a projection of the stack sections that contained the most anterior scleral canal. By doing the projection based on the anterior portion of the canal, where the canal is narrowest^36,37^, we avoided potential artifacts due to the choice of projection direction. All histological markings on projected images were also cross-checked for consistency with the registered 3D stacks.

### Fixative effects

Region-dependent effects of formalin fixation were determined as percentage changes in area of cornea, sclera, PPONH, and scleral canal across three time points: fresh, fixed for 1 minute, and fixed for 24 hours. Directional-dependent, or anisotropic, effects of formalin fixation were determined as percentage change in length, diameter, and thickness of the equatorial sclera, PPONH, and scleral canal across the three time points. The dimensions of scleral samples were not standardized with a template. Thus, all changes due to fixation were tested relative to the same-sample fresh measures to account for the variations between manually-cut samples.

### Sectioning effects

We further compared the area and circularity of the PPONH, as well as area, diameters and aspect ratio of the scleral canal sizes after the histological processing with those from fresh tissues to evaluate the full processing effect, and with those from 24-hour fixation to isolate the effect of the tissue processing that is not due to fixation.

### Statistical analysis

Statistical analysis was performed in SPSS (IBM Corp., Armonk, NY) using one-way repeated Analysis of Variance (ANOVA). Specifically, for fixative effects on 14 outcome parameters, repeated measurements were compared across three time points, fresh, fixed 1 minute, fixed 24 hours. For sectioning effects on 6 outcome parameters, repeated measurements were compared across four time points, fresh, fixed 1 minute, fixed 24 hours. Significant ANOVA results were followed up with paired t-tests. Significant levels were initially set to 0.05, and then adjusted for multiple comparisons using Bonferroni corrections. Thus, significance was declared at p = 0.0025 for ANOVA and p = 0.0056 for paired t-tests.
